# Timing and Outcomes of Vitreoretinal Surgery after Traumatic Retinal Detachment

**DOI:** 10.1155/2016/4978973

**Published:** 2016-11-23

**Authors:** Molly Orban, Yasmin Florence Khodeja Islam, Luis J. Haddock

**Affiliations:** ^1^University of Central Florida College of Medicine, Orlando, FL, USA; ^2^University of Florida College of Medicine, Gainesville, FL, USA; ^3^Bascom Palmer Eye Institute at Palm Beach Gardens, Palm Beach Gardens, FL, USA

## Abstract

Traumatic retinal detachments are a significant cause of morbidity. There are currently no evidence-based guidelines on the appropriate time to perform vitreoretinal surgery to repair a traumatic retinal detachment. Early intervention, within seven days of the inciting trauma, may decrease proliferative vitreoretinopathy and postoperative endophthalmitis. Later intervention may yield a reduced risk of inflammation and hemorrhage, particularly in cases of concomitant open globe injuries. This article reviews the literature on the management of retinal detachments associated with ocular trauma from the years 2006 to 2016. Particular focus was placed on the timing of surgery, concomitant open globe injury, anatomical success rates, visual acuity, and complication rates. In this review, anatomical success was not significantly related to timing of intervention when compared between early and delayed intervention in eyes with and without concomitant open globe injuries. Visual acuities postoperatively varied widely despite timing of intervention due to the large variation in mechanism and extent of ocular injuries. Proliferative vitreoretinopathy was a common complication. Preliminary data indicate that endophthalmitis rates may be lower when early vitreoretinal surgery is performed. There is insufficient data to conclude whether early or delayed surgery leads to improved outcomes, highlighting the need for further research in this domain.

## 1. Introduction

Ocular trauma is the leading cause of decreased visual acuity in young adults [1]. 0.8% of retinal detachments (RDs) in the general population are secondary to trauma [2]. Most of these traumatic RDs are in young patients with work-related trauma [3, 4]. There is a general consensus that vitrectomy is the appropriate surgery for most traumatic RDs, but other interventions may include scleral buckle, laser, retinopexy, or a combination of these based on the individual patient. The timing to intervention, however, remains controversial [5, 6].

In addition to repairing the retinal detachment, the benefit of early intervention with vitrectomy is to remove the maximum amount of vitreous, thus eliminating the depot for inflammation to settle and epiretinal membranes to form. Some authors postulate that early intervention decreases postoperative proliferative vitreoretinopathy (PVR), a complication that can lead to redetachment and has been found to be more common in eyes that have suffered trauma than in nontraumatic RD [7]. Others contend for delayed intervention, allowing time for the eye to recover, a complete posterior vitreous detachment to develop, and the PVR process to settle, thus making the surgery less complicated. They also maintain that there is a decreased risk of severe hemorrhage when operating several days after the inciting trauma, particularly in eyes with prior open globe injuries. In delayed intervention, primary ocular repair, if needed, is often done within hours of the injury to stabilize the eye, while vitreoretinal surgery takes place up to one month later [6]. This review analyzes recent publications and research in the management and outcomes of traumatic RDs to suggest updated guidelines for the management of this condition, with particular attention as to whether vitreoretinal surgery should occur within the first 7 days after injury or later.

## 2. Methods

The following search terms were entered into http://www.pubmed.gov/ (US National Library of Medicine, National Institutes of Health): “(Retinal Detachment/statistics and numerical data” [Mesh] OR “Retinal Detachment/surgery” [Mesh] OR “Retinal Detachment/therapy” [Mesh]) AND “Craniocerebral Trauma” [Mesh]. Results were limited to the research studies conducted in humans within the past ten years (2006–2016) that are written in English. This search yielded 70 articles. Two independent reviewers analyzed all 70 articles and agreed that 24 of these articles were pertinent to this review. After extensive review of these 24 articles, it was mutually decided that 9 had data suitable to this review. Articles were excluded if they did not discuss traumatic retinal detachment, if they did not separate outcome measures based on whether patients had retinal detachment versus other types of retinal pathology, or if they did not involve surgical intervention for retinal detachment. For individual consideration of the impact of time to intervention, articles were only included if they reported outcome measures individually for each patient or stratified patients by timing to intervention.

Outcome measures studied included percentage of patients with improved visual acuity postoperatively and percentage of patients with attached retina at follow-up. Follow-up period was 3 months or greater for each study. A Pearson Chi-square test was used to determine whether timing to intervention resulted in a significantly different number of patients with attached retina at follow-up. The timing variable for each study was coded in two groups, one with patients receiving intervention within 7 days including those who had concomitant open globe repair and RD repair and the other with patients receiving intervention greater than 7 days after presentation. All studies fit into these two categories except Ehrlich and Polkinghorne [8], which reported a mean time to intervention of 22.4 days, which was coded in the timing category as greater than 7 days to intervention. *P* values of less than 0.05 were considered statistically significant, and hazard ratios were conducted with a 95% confidence interval by the Cox proportional-hazard analysis. Statistical analysis was conducted using Statistical Package for the Social Sciences (SPSS) version 22.0 (IBM; Chicago, IL). Type of intervention and complications were reported for each study which included this data.

## 3. Results

### 3.1. Timing to Intervention

Most patients underwent primary closure promptly if necessary for open globe injury, but time to vitreoretinal intervention for retinal detachment varied. Of the 9 included articles, 5 provided information on timing to intervention (Table 1). Ehrlich and Polkinghorne reported a mean time to vitreoretinal intervention of 22.4 days (range 3–86 days) among a total of 19 adult patients and was therefore analyzed as greater than seven days before intervention [8]. Among the five articles, 171 patients in total underwent intervention within 7 days of diagnosis of trauma related RD, and 28 patients underwent intervention greater than 7 days after diagnosis of RD. Of those with intervention within 7 days, 88 of those had vitreoretinal surgery 48 hours or less after diagnosis of RD [9, 10]. Nashed et al. reported on 88 that underwent retinal detachment repair at the same time as open globe repair within 8 hours of initial presentation [5]. Some of the patients, including those reported by Wang et al., were pediatric patients (Table 1). These patients were all included in the analysis. Of note, none of these studies except Nashed et al. [5] identified and separated patients by the timing of open globe injury repair, whether it was prior to vitreoretinal surgery or concomitant with it.

### 3.2. Attached Retina at Follow-Up

A Chi-square test of independence determined that the relationship between the timing of intervention and the number of patients with an attached retina at follow-up was not significant; *χ* ^2^ (1, *N* = 199) = 0.216, *p* = 0.642. The effect size showed phi = 0.033. Results from each study individually can be found in Table 2. No other factors such as concomitant open globe or type of surgical intervention were found to be statistically significant in regard to subsequent anatomic success at follow-up.

### 3.3. Visual Acuity

A comparison of preintervention and postintervention best corrected visual acuity (BCVA) can be found in Table 3. Two of the studies included in our analysis provided visual acuities before and after surgery for each patient, and thus we compared the preoperative and postoperative visual acuities to determine if their vision improved, was stable, or deteriorated after intervention (Table 4) [8, 10]. The surgical interventions in these two studies included both vitrectomy and scleral buckle placement. In 54% of patients, visual acuity (VA) was improved after surgical intervention, whereas 36% of patients had a worsened VA after surgery. 10% of patients had a stable VA [8, 10]. Up to 32% of patients ultimately had no light perception, while up to 13% of patients achieved 20/20 VA [10] (Table 3). In Sheard et al.'s study, 65% of pediatric patients receiving scleral buckle had improved VA after intervention, 18% had worsened VA, and 18% were stable. Amongst the vitrectomy patients in the same study, 36% had improved VA after intervention, 36% had worsened VA, and 29% were stable [13].

### 3.4. Type of Intervention

Type of vitreoretinal intervention was reported for a total of 294 patients among the 9 included studies (Table 5). 159 patients underwent vitrectomy alone, 32 had scleral buckle placement alone, and 34 had both vitrectomy and placement of a scleral buckle. Of note, Rouberol et al. (50 patients) and Lesniak et al. (19 patients) stated that patients had vitrectomy with or without scleral buckle. The exact number of patients with adjuvant use of scleral buckle during vitrectomy was not reported in either of these articles; therefore these treatment counts are not included in the totals above. Silicone oil tamponade was used in a total of 131 patients. There is insufficient data to assess whether the type of surgical intervention had an impact on either anatomical success or postoperative visual acuity.

### 3.5. Complications

Complication rates following vitreoretinal surgery for traumatic retinal detachment in these studies are detailed in Table 6. The vast majority of phakic eyes developed cataracts after surgical intervention [9, 11, 17]. Development of secondary glaucoma was also a significant complication and in some cases required surgical intervention [10, 15]. Multiple investigators found that patients developed phthisis bulbi, with up to 30% of patients experiencing this complication in certain studies [5, 8–10, 12]. PVR was a significant finding postoperatively, as it was found in up to 56% of patients [5, 8, 12, 15].

## 4. Discussion

This review analyzes the available articles over the last 10 years since the use of small gauge vitrectomy which included a diverse population of patients with traumatic retinal detachment, including both pediatric and adult patients. Notably, the mechanism of ocular injury, the extent of injuries, timing of RD development, and surgical management varied significantly between patients in the available studies.

### 4.1. Predictive Factors of Postoperative Retinal Attachment

In our review, reattaching the retina within 7 days after trauma did not significantly affect the likelihood that the retina would remain attached at follow-up, nor did waiting over 7 days before vitreoretinal surgery affect anatomical success rates. For all studies, this was assessed at more than three months after the surgery. This review highlights a lack of literature regarding this topic in regard to the appropriate intervention in these cases with or without the presence of open globe injury, suggesting the need of additional studies to help identify these factors. There is insufficient data to assess if the type of vitreoretinal intervention affects anatomical success or postoperative visual prognosis.

The available cases in the literature are limited and also represent a large mix of patients who had different mechanisms and extent of ocular trauma leading to many confounding factors in our analysis. Current recommendations are trending towards earlier intervention to reduce the risk of extension of the retinal detachment and increased proliferation of the epiretinal membranes. Further analysis of these cases should be performed in order to develop better guidelines as to the ideal time to repair these traumatic retinal detachments.

### 4.2. Predictive Factors of Postoperative Visual Acuity

There is insufficient data to conclude whether timing of intervention affects postoperative visual acuity. However, this is not the only factor affecting visual potential because regardless of what time vitreoretinal surgery is performed, postoperative VA varies widely. This makes it difficult to counsel patients about their expected visual outcome when they have traumatic retinal detachments.

This inability to accurately predict postoperative VA has led several investigators to study what factors influence final VA. Multiple studies support that a poorer VA at presentation predicts a poorer final visual outcome [9, 12, 18]. Attachment of the macula at presentation was associated with an improved visual outcome [9, 10]. Developing endophthalmitis conferred an inferior visual outcome in one study [18] but was not shown to have the same effect in another [14]. Recurrent retinal detachment predicted a worse visual outcome [10], as did total RD on presentation, retention of silicone oil in the eye, and development of PVR [14]. The following factors were not shown to significantly influence visual outcome: an open globe injury, previous ocular surgery, lens injury, number and severity of retinal tears, presence of vitreous hemorrhage [9, 14, 16], need for multiple vitreoretinal procedures, and whether the patient received scleral buckling or PPV [9, 12, 14].

Most cases of redetachment after surgery were secondary to proliferative vitreoretinopathy [9, 14]. Wang et al. found that, in pediatric patients with traumatic RD, age, sex, location of injury, time between injury and RD, vitreous hemorrhage, and lens status did not predict anatomical success rate. However, poor preoperative VA (LP or worse), presence of PVR grade C or worse, detachment of the macula, total RD, and vitrectomy (as opposed to scleral buckle) are associated with a worse anatomical success rate [10].

An important point to consider is that RD does not always occur immediately after trauma. Other investigators have studied which patients are more likely to develop a retinal detachment after traumatic globe injury. Stryjewski et al. studied open globe injuries and found that a visual ability to count fingers (or worse), injury involving the sclera posterior to the limbus, and presence of vitreous hemorrhage all correlated with an increase rate of developing subsequent traumatic RD. They subsequently created a scoring system to predict the likelihood of a retinal detachment after open globe injury based on the presence of these characteristics [19]. However their analysis did not make any recommendations regarding the management of these cases once they develop retinal detachments. Pimolrat et al. also found that, in patients who require pars plana vitrectomy (PPV) after open globe injury, the presence of a rapid afferent pupillary defect was significantly associated with a worse visual acuity after vitrectomy. However, only 32% of the patients in this study had a RD, limiting its applicability to patients who have already developed a RD [20].

### 4.3. Complications of Surgical Intervention for Traumatic Retinal Detachment

While surgical intervention for traumatic retinal detachment can be vision-saving, it is not without its risks, as detailed in Table 5. PVR is a major concern after surgery for RD, with up to 56% of patients developing this complication [15]. As discussed previously, PVR is a significant risk factor for recurrent retinal detachment, so this complication is of particular importance. In a study of 28 patients treated with cryobuckle only for retinal detachment due to retinal dialysis, only 1 (3.6%) developed PVR [16]. This may indicate that cryobuckle alone is associated with a decreased risk of PVR development. However, this study had a small population and was limited to only patients with traumatic RD with retinal dialysis, which is often amenable to repair with buckle only. This is in contrast to most other traumatic RDs which have limited success with buckle-only repair. Further studies are necessary to explore this relationship. Endophthalmitis rates were as low as 3.4% when surgical intervention for RD repair was performed within 8 hours [5], whereas when timing of surgery is not taken into account, other authors report that ocular trauma resulted in endophthalmitis in up to 13% of cases [18]. These results suggest that other important outcome measures are also related to timing of vitreoretinal intervention, and further studies delineating the risks and benefits of early surgical intervention should be pursued. Traumatic retinal detachments are challenging and decisions should be individualized; however further analysis to help identify which surgical techniques improve outcome should be undertaken.

## 5. Conclusion

There is insufficient data to assess if early vitreoretinal surgery to repair traumatic RDs leads to significantly higher reattachment rates at long-term follow-up and improved visual outcomes for patients. Common adverse outcomes after vitreoretinal surgery for traumatic RD include PVR, cataracts, secondary glaucoma, phthisis bulbi, and endophthalmitis. Preliminary studies indicate that endophthalmitis rates may be lower when early vitreoretinal surgery is performed. The information in this review suggests it is too soon to determine if vitreoretinal surgery for repair of traumatic retinal detachment should be performed within 7 days of the inciting event, and further studies should focus on prospectively determining the effect of timing to intervention particularly in cases of concomitant open globe injuries on clinical outcome variables and complication rates.

## Figures and Tables

**Table 1:** Time to and type of intervention.

Study	Time to intervention	Type of intervention	Number of patients
Nashed et al. [5]	<8 hours	Vitrectomy with silicone oil within 8 hours of presentation	88

Ehrlich and Polkinghorne [8]	>7 days	Small-gauge vitrectomy ± scleral buckle ± silicone oil tamponade	19

Rouberol et al. [9]	≤7 days	94% underwent cryotherapy 82% had a scleral buckle 76% underwent PPV	50

Wang et al. [10]	≤7 days	88% underwent PPV 70% had a scleral buckle placed	33

Sisk et al. [11]	>7 days	78% PPV and scleral buckle 11% PPV alone 11% scleral buckle alone	9

**Table 2:** Time to intervention versus percent attached at follow-up.

Study	Timing	Number not attached at F/U (%)	Number attached at F/U (%)	Total patients studied
Nashed et al. [5]	≤7 days	33 (37.5%)	55 (62.5%)	88

Ehrlich and Polkinghorne [8]	>7 days	6 (31.6%)	13 (68.4%)	19

Rouberol et al. [9]	≤7 days	12 (24%)	38 (76%)	50

Wang et al. [10]	≤7 days	24 (72%)	9 (27%)	33

Sisk et al. [11]	>7 days	4 (44%)	5 (55%)	9

**Table 3:** Visual Acuity after Traumatic Retinal Detachment.

Author	Number of patients	Initial (preintervention) BCVA	Postintervention BCVA	Comments
Nashed et al. [5]	88	2.3%: **≥**20/5016%: >20/800	8%: ≥20/5050%: >20/800	36% of patients who received a retinectomy had a VA of >20/800 after intervention

Ehrlich and Polkinghorne [8]	19	5%: ≥20/5022%: <20/50 & ≥20/200 5%: <20/200 & ≥20/400 69%: <20/400	21%: ≥20/50 5%: <20/50 & ≥20/200 11%: <20/200 & ≥20/400 63%: <20/400	63% had improved VA 21% had worsened VA 16% were stable

Rouberol et al. [9]	50	42%: **≥**20/200	38%: ≥20/4080%: ≥20/200	

Wang et al. [10]	31	3%: ≥20/509%: <20/50 & ≥20/200 87%: <20/400	28%: ≥20/509%: <20/50 & ≥20/200 3%: <20/200 & ≥20/400 58%: <20/400	These were all pediatric patients Only patients who have both a preintervention and postintervention BCVA are included in this chart 48% has improved VA 45% had worsened VA 6% were stable

Eliott et al. [12]	1	20/30	20/20	Pediatric patients

Sheard et al. [13]	47	Median for scleral buckle patients: 20/120 Median for vitrectomy patients: CF	Median for scleral buckle patients: 20/80Median for vitrectomy patients: CF	These were all pediatric patients

Zhang et al. [14]	9	LP (average)	Between LP and HM	No statistical difference between preintervention and postintervention VA

Lesniak et al. [15]	28	LP (average)	20/4000 (average)	Pediatric patients with open globe injuries

**Table 4:** Time to intervention versus percent with improved or stable visual acuity.

Study	Timing	Percent with improved or stable VA	Number with improved/stable VA	Total patients studied
Ehrlich and Polkinghorne [8]	>7 days	79	15	19

Wang et al. [10]	≤7 days	54	18	33

**Table 5:** Type of intervention.

Study	Type of intervention	Number of patients
Nashed et al. [5]	Vitrectomy	88
Ehrlich and Polkinghorne [8]	Vitrectomy	16
	Vitrectomy + buckle	3
Rouberol et al.^*∗*^ [9]	Vitrectomy +/− buckle	50
Wang et al. [10]	Buckle	4
	Vitrectomy + buckle	19
	Vitrectomy	10
Sisk et al. [11]	Vitrectomy	1
	Vitrectomy + buckle	7
	Buckle	1
Eliott et al. [12]	Cryopexy + radial sponge	1
Sheard et al.^*∗∗*^ [13]	Vitrectomy	30
	Buckle	17
Lesniak et al.^*∗∗∗*^ [15]	Vitrectomy +/− buckle	19
James et al. [16]	Vitrectomy	14
	Cryobuckle	9
	Vitrectomy + cryobuckle	5

Treatment breakdown for each study included:

^*∗*^9 patients underwent no vitreoretinal intervention: 4 primary enucleation, 3 secondary enucleation, 1 no treatment due to phthisis, and 1 was lost to follow-up.

^*∗∗*^This study also included another 14 patients who underwent vitrectomy for retinal pathology that was not an RD; these patients are not included above.

^*∗∗∗*^Unclear how many patients underwent vitrectomy alone versus vitrectomy + buckle.

**Table 6:** Complications after surgery for traumatic retinal detachment.

Author	Number of eyes	Type of intervention	Timing to intervention	Complications	Comments
Nashed et al. [5]	88	Vitrectomy with SO within 8 hours of presentation	Within 8 hours	49% retained SO 44%: PVR 8%: phthisis bulbi 3.4%: endophthalmitis	

Ehrlich and Polkinghorne [8]	19	Small-gauge vitrectomy ± SB ± SO tamponade	Mean: 22.4 d	21% had OHTN that responded to topical treatment 11% had hypotony (≤6 mm Hg) 5% developed PVR 5% developed phthisis bulbi	

Rouberol et al. [9]	50	94% underwent cryotherapy 82% had a SB 76% underwent PPV	Within 7 days	8% retained SO 6%: a secondary cataract 4%: an ERM 2% developed SO emulsification in the AC 2% developed phthisis bulbi	25 patients had open globe injuries, while 25 had closed globe injuries.

Wang et al. [10]	33	88% underwent PPV 70% had a SB placed	Within 7 days	30%: phthisis bulbi 18%: band keratopathy 15%: rubeosis 6%: VH 3%: secondary glaucoma 3%: siderosis	These were all pediatric patients.

Sisk et al. [11]	9	78% PPV and SB 11% PPV alone 11% SB alone	Varied 6/9 cases were chronic RD, likely delayed presentation	89% developed a cataract 56% developed secondary glaucoma after SO placement 56% developed PVR 56% developed complications from SO, such as emulsification blocking the visual axis 44% had recurrent RD	These patients all had repeated head trauma from self-injurious behavior. Some of these RDs were chronic.

Kolomeyer et al. [17]	41	PPV with 360° retinectomy	Varied	100% of phakic eyes developed a cataract 39%: PVR 32%: significant retinal hemorrhage intraoperatively 17%: corneal decompensation 17%: hypotony 17%: preretinal or retinectomy hemorrhage 15%: hyphema 15%: phthisis bulbi 5%: subretinal Perfluoron droplets	Only 63% of patients had traumatic RD.

James et al. [16]	28	100% had cryobuckle placement		46%: buckle removal 25%: buckle exposure 18%: strabismus 11%: infection related to the buckle	

SO: silicone oil, OHTN: ocular hypertension, ERM: epiretinal membrane, SB: scleral buckle, AC: anterior chamber, VH: vitreous hemorrhage, RD: retinal detachment, PVR: proliferative vitreoretinopathy.
